# Apo- and holo-transferrin differentially interact with hephaestin and ferroportin in a novel mechanism of cellular iron release regulation

**DOI:** 10.1186/s12929-023-00934-2

**Published:** 2023-06-06

**Authors:** Stephanie L. Baringer, Kondaiah Palsa, Vladimir S. Spiegelman, Ian A. Simpson, James R. Connor

**Affiliations:** 1 Department of Neurosurgery, Penn State College of Medicine, 500 University Drive, Hershey, PA 17033 USA; 2 Department of Pediatrics, Penn State College of Medicine, Hershey, PA USA; 3 Department of Neural and Behavioral Sciences, Penn State College of Medicine, Hershey, PA USA

**Keywords:** Blood–brain barrier, Iron, Transferrin, Hepcidin, Ferroportin, Hephaestin

## Abstract

**Background:**

Apo- (iron free) and holo- (iron bound) transferrin (Tf) participate in precise regulation of brain iron uptake at endothelial cells of the blood–brain barrier. Apo-Tf indicates an iron-deficient environment and stimulates iron release, while holo-Tf indicates an iron sufficient environment and suppresses additional iron release. Free iron is exported through ferroportin, with hephaestin as an aid to the process. Until now, the molecular mechanisms of apo- and holo-Tf influence on iron release was largely unknown.

**Methods:**

Here we use a variety of cell culture techniques, including co-immunoprecipitation and proximity ligation assay, in iPSC-derived endothelial cells and HEK 293 cells to investigate the mechanism by which apo- and holo-Tf influence cellular iron release. Given the established role of hepcidin in regulating cellular iron release, we further explored the relationship of hepcidin to transferrin in this model.

**Results:**

We demonstrate that holo-Tf induces the internalization of ferroportin through the established ferroportin degradation pathway. Furthermore, holo-Tf directly interacts with ferroportin, whereas apo-Tf directly interacts with hephaestin. Only pathophysiological levels of hepcidin disrupt the interaction between holo-Tf and ferroportin, but similar hepcidin levels are unable to interfere with the interaction between apo-Tf and hephaestin. The disruption of the holo-Tf and ferroportin interaction by hepcidin is due to hepcidin’s ability to more rapidly internalize ferroportin compared to holo-Tf.

**Conclusions:**

These novel findings provide a molecular mechanism for apo- and holo-Tf regulation of iron release from endothelial cells. They further demonstrate how hepcidin impacts these protein–protein interactions, and offer a model for how holo-Tf and hepcidin cooperate to suppress iron release. These results expand on our previous reports on mechanisms mediating regulation of brain iron uptake to provide a more thorough understanding of the regulatory mechanisms mediating cellular iron release in general.

**Supplementary Information:**

The online version contains supplementary material available at 10.1186/s12929-023-00934-2.

## Background

Regulation of iron uptake at the blood–brain barrier (BBB) is crucial for proper brain function. Detrimental alterations in brain iron homeostasis can lead to a variety of neurological conditions, including but not limited to neurodegenerative diseases (Alzheimer’s disease, Parkinson’s disease, and amyotrophic lateral sclerosis) [1] and Restless legs syndrome [2]. Our group and others have shown that endothelial cells (ECs) of the BBB serve as reservoirs for iron before it is subsequently released into the extracellular fluid of the brain. Moreover, this release is regulated by levels apo (iron free)- and holo (iron bound)-transferrin (Tf) [3–7] in extracellular fluid. Using both in vitro [3, 4, 6] and in vivo [7] models, we have shown that increasing the ratio of apo- to holo-Tf, reflecting an iron deficient environment, stimulates iron release from ECs, whereas elevated holo-Tf relative to apo-Tf, reflecting an iron-replete environment, suppresses iron release. This feedback mechanism allows for regional specificity of iron uptake based on regional iron consumption and metabolic needs [8, 9].

Free iron is released from cells, including ECs, through ferroportin (Fpn), the only know iron exporter. Fpn function is aided by a number of proteins, including hephaestin (Heph) [10, 11], a ferroxidase that converts released ferrous (Fe2+) to ferric (Fe3+) iron. Heph is required for both the stability of Fpn in the plasma membrane and the efflux of iron through Fpn [10, 11]. Inversely, Fpn can be inhibited by hepcidin [12], a pro-inflammatory peptide hormone, primarily secreted by the liver [13] and in small amounts by astrocytes [14]. When hepcidin binds to Fpn, Fpn is ubiquitinated for internalization and subsequent degradation [12, 15]. Simpson et al*.* found that, in addition to iron release, holo-Tf also decreases Fpn protein in EC culture models of the BBB [6] but the mechanism was unclear. Conversely, it has been proposed that apo-Tf participates in interactions with ferroxidases such as Heph and ceruloplasmin to facilitate iron release [16–18]. In the present study, we have determined the differential interactions that apo- and holo-Tf have with Fpn and Heph to control iron release. Moreover, we demonstrate the impact that hepcidin can have on these interactions. These results provide significant novel insights not only into the regulatory mechanism of iron release into the brain but are likely relevant to cellular iron release in general.

## Methods

### Cell culture

Human endothelial-like cells (ECs) were differentiated from ATCC-DYS0100 human iPSCs as described previously [19, 20]. Briefly, iPSCs were seeded onto a Matrigel-coated plate in E8 medium (Thermo Fisher Scientific, 05990) containing 10 µM ROCK inhibitor (Y-27632, R&D Systems, 1254) at a density of 15,000 cells/cm^2^. The iPSCs differentiation was initiated by changing the E8 medium to E6 medium (Thermo Fisher Scientific, A1516401) after 24 h. E6 medium was changed daily up to 4 days before switching to human endothelial serum free medium (hESFM) (Thermo Fisher Scientific, 11111) supplemented with 10 nM bFGF (Fibroblast growth factor, Peprotech, 100-18B), 10 µM all-trans retinoic acid (RA, Sigma, R2625), and 1% B27 (Thermo Fisher Scientific, 17504–044). After 48 h of no medium changes, cells were harvested and replated onto Transwell filters coated with collagen IV and fibronectin. Twenty-four hours after replating, bFGF and RA were removed from the medium to induce barrier phenotype. HEK 283 cells were maintained in Dulbecco's modified Eagle's medium (DMEM, Gibco, 11965–084) and supplemented with 10% FBS and 1% penicillin–streptomycin (Gibco, 15070063).

### Plasmid and transfection

HEK 293 cells were seeded at a density of 7 × 10^4^ cell/cm^2^ in a 6-well plate. The following day, the cells were transfected with 1 μg/well of the HA-tagged Fpn plasmid (Vector Builder, VB220407-1185gaa, Additional file 1: Fig. S1) using Lipofectamine™ 3000 Transfection Reagent (Invitrogen, L3000001).

### Co-immunoprecipitation

In order to remove any exogenous Tf, the media was replaced with DMEM containing no FBS or B27 24 h before the start of experiments. Cells were exposed to apo- or holo-Tf (Sigma, T1147 and T4132) for 10 min and then washed on ice with cold PBS twice. Chilled 100 μl Co-IP lysis buffer (20 mM Tris HCl, pH 8, 137 mM NaCl, 10% glycerol, 1% Triton x-100, and 2 mM EDTA) was added to each well. Cells were collected and incubated with rotation for 30 min at 4 °C. Cell solutions were centrifuged at 14,000×*g* for 20 min at 4 °C. Supernatant was collected, and protein estimation was performed using Pierce BCA Protein Assay Kit (Thermo, 23227). Approximately 1 mg of protein was used for Co-IP using anti-HA magnetic beads (Thermo, 88837) or Protein G magnetic beads (Thermo, 10003D) complexed with anti-Heph antibody (Santa Cruz, SC-365365) according to manufacturer’s instructions [21]. Briefly, magnetic beads were washed twice with PBS before adding lysates. The bead and lysate solutions were incubated with rotation for 30 min at room temperature. After washing with PBS, protein was eluted from beads by resuspending in non-reducing sample buffer and boiling at 90 °C for 10 min. Magnet was used to isolate the magnetic beads from the protein solution, which was then reduced using 2 M DTT and then loaded for immunoblotting.

### Proximity ligation assay (PLA)

PLA is a technique that precisely demonstrates if two proteins directly interact with one another. When two proteins are in sufficiently close proximity to interact, the secondary oligomer probes ligate together, allowing for the amplification of the oligomers and resulting in a fluorescent signal. PLA was performed using a Duolink assay kit (Sigma-Aldrich, DUO92013) according to the manufacturer's instructions [22]. Chamber slides (Falcon, 354108) were coated with poly-d-lysine 2 h before HEK 293 cells were culture on the slides at a density of 15,000 cell/cm^2^. In order to remove an exogenous Tf, 24 h later the media was replaced with DMEM containing no FBS. Cells were exposed to apo- or holo-Tf (Sigma, T1147 and T4132) for 10 min and then washed to procced with PLA. Primary antibodies used were the following: myelin basic protein 1 (MBP1, Abcam, ab22460, 1:500), ferritin (Abcam, ab77127, 1:500), Tf (ProteinTech, 66161-1, 1:500), TfR (Cell Signaling, 13208S, 1:500), Tf (Abcam, ab82411, 1:500), Fpn (gift from M. Knutson, 1:500), and Heph (Santa Cruz, SC-365365, 1:500). Positive and negative controls used for assay optimization can be found in Additional file 1: Fig. S2. Imaging and analysis were performed using Revolve R4 microscope (Echo). The integrated density was calculated by summing the pixels from PLA signal and dividing by the field of view area. The integrated density of background from negative controls were subtracted from these values. To determine the integrated density per cell, this was then divided by the number of cells in the field of view. A minimum of three images were taken in different regions of the slides and then averaged for a single biological replicate. Image brightness was uniformly increased for the purposes of publication but not for quantification.

### Membrane protein isolation

Cells were washed with PBS three time before incubating with 200 μl digitonin buffer (20 mM Tris–HCl, 250 mM sucrose, 0.007% digitonin, 1 × protease inhibitor cocktail) [23]. Cells were gently lifted from the plate and collected in chilled glass mini homogenizers. Once homogenized, samples were spun at 1500×*g* for 10 min. The pellet was reserved and the supernatant was spun again at 10,000×*g* for 10 min. The resulting pellet was combined with the pervious pellet and resuspended in RIPA buffer and 1 × protease inhibitor cocktail. After immunoblotting was performed on the samples, the membranes were stained for total protein content using Ponceau S staining solution (Thermo, A40000279) to use as a loading control.

### Immunoblotting

Samples were loaded onto a 4–20% Criterion TGX Precast Protein Gel (Bio-Rad) [7]. Protein was transferred onto a nitrocellulose membrane and probed for Fpn (Alpha Diagnostics, MTP11-S, 1:1000), DMT1 (Millipore, ABS983, 1:1000), Heph (Santa Cruz, SC-365365, 1:1000), TfR (Santa Cruz, sc-65882, 1:250), Tf (Abcam, ab82411, 1:1000), HA tag (Invitrogen, MA5-27915, 1:1000), ubiquitin (Protein Tech, 10201–2-AP, 1:1000) or cyclophilin B (Abcam, ab16045, 1:1000) as a loading control. Corresponding secondary antibody conjugated to HRP was used (1:5000, GE Amersham) and bands were visualized using ECL reagents (Perkin-Elmer) on an Amersham Imager 600 (GE Amersham). Cellular lysate samples were normalized to cyclophilin B protein as a loading control, and then subsequently normalized to an untreated control sample within each experiment. Membrane protein samples were stained with Ponceau S and normalized to total protein as a loading control.

### Statistical analysis

Statistical analyses were performed using Prism 9.2 software (Graphpad Software Inc.). Data from at least three independent biological replicates were averaged and are expressed as the mean ± standard error of the mean (SEM). One-way ANOVA with Tukey post-hoc analysis, two-way ANOVA with Sidak’s post hoc analysis, or unpaired t-tests were used to evaluate for statistical significance where appropriate. A p-value < 0.05 was considered significant.

## Results

### Holo-Tf decreases Fpn levels through the Fpn degradation pathway

**Fig. 1:**
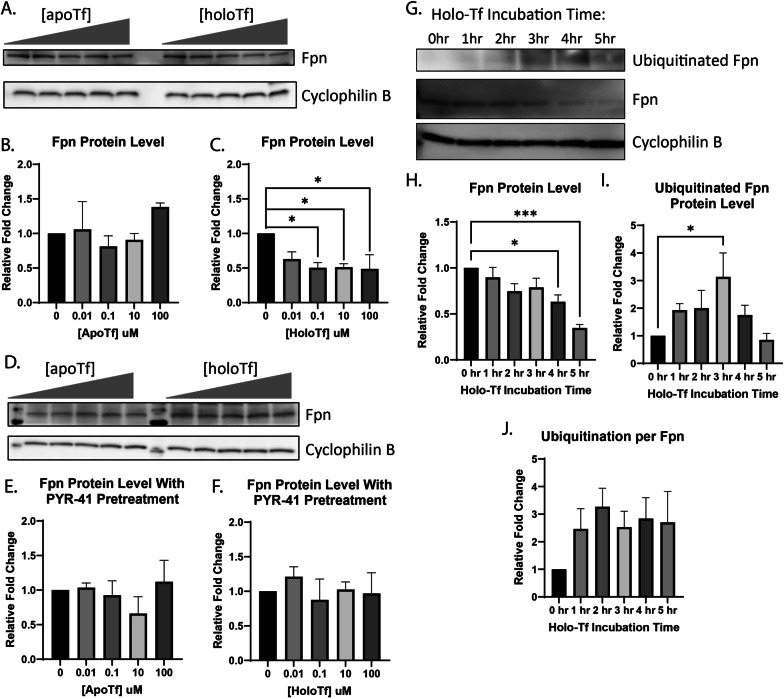
Modulation of Fpn protein levels in ECs by holo-Tf. iPSC-derived ECs were cultured on bi-chamber plates, incubated with apo- or holo-Tf in the basal chamber, and collected after 8 h for immunoblotting. Fpn protein levels were normalized to cyclophilin B as a loading control. All quantifications were further normalized to untreated control to account for cell count variability. Holo-Tf decreased Fpn protein levels by 50% at concentrations as low as 0.1 μM, while apo-Tf did not (**A**–**C**). Holo-Tf-mediated internalization and degradation of Fpn was inhibited by a ubiquitination inhibitor, PYR-41, (**D**–**F**) confirming that holo-Tf’s decreases Fpn through the established degradation pathway. ECs were incubated with 0.25 μM holo-Tf to observe the ubiquitination and internalization of Fpn over time. After 1 h, ubiquitination of Fpn was detected, with a maximal effect at 3 h (**G**–**J**). By 5 h, 50% of Fpn is internalized with continuous ubiquitination per Fpn present. n = 3 for all experiments, means of biological replicates ± SEM were evaluated for statistical significance using one- way ANOVA with Tukey’s posttest for significance. *p < 0.05, ***p < 0.001

In the first series of experiments, we examined the effects of apo- and holo-Tf on the cellular levels of Fpn by incubating iPSC-derived ECs with increasing concentrations of either apo- or holo-Tf in hESFM for 8 h. ECs were cultured onto Transwell inserts and apo- or holo-Tf was placed in the basal chamber to represent the brain-side. The ECs were collected and probed for various iron transport proteins. Incubations with holo-Tf decreased Fpn protein levels by 50% at concentrations as low as 0.1 μM (*p < 0.05, Fig. 1A) whereas apo-Tf had no impact on Fpn (Fig. 1A). Other iron transport proteins, such as Heph, DMT1, and TfR, were relatively unchanged with incubations of apo- or holo-Tf (Additional file 1: Fig. S3).

The degradation pathway for Fpn involves ubiquitination by E1 ubiquitin ligase, resulting in the internalization and degradation of Fpn [15, 24]. To determine if this established degradation pathway was the cause of the decreased Fpn induced by holo-Tf, we pretreated ECs with 50 μM PYR-41, an E1 ubiquitin ligase inhibitor, before exposure to either apo- or holo-Tf. The use of 50 μM PYR-41 to prevent Fpn ubiquitination has been demonstrated previously [24]. The inhibition of Fpn ubiquitination resulted in a mitigation of holo-Tf’s decrease of Fpn (Fig. 1D), while apo-Tf continued to have no impact on Fpn levels (Fig. 1D). In order to confirm the ubiquitination inhibition by PYR-41 in our experiments, we exposed ECs to 500 nM of hepcidin (standard concentration in the literature [12, 15, 24]) following pretreatment with 50 μM PYR-41 for 30 min (Additional file 1: Fig. S3). Controls were either solely exposed to hepcidin or PYR-41. As expected, hepcidin alone increased Fpn ubiquitination and PYR-41 pretreatment prevented this increase (Additional file 1: Fig.S 3).

To further confirm that holo-Tf induces the ubiquitination of Fpn and observe the timing of Fpn degradation, we incubated ECs in Transwell inserts with 0.25 μM holo-Tf (physiological level in CSF [25]) in the basal chamber for intervals of 1 h before collecting the cells and probing for ubiquitinated protein and Fpn. In a time-dependent manner, Fpn levels decrease over time with incubation of holo-Tf (Fig. 1G). After 5 h of holo-Tf incubation, Fpn levels have decreased to about 50% (***p < 0.001, Fig. 1H). Furthermore, the levels of ubiquitinated Fpn increase over time, with a maximal effect at 3 h (*p < 0.05, Fig. 1I). Because Fpn is degraded over time, we further calculated the extent of ubiquitination relative to the amount of Fpn (Fig. 1J). The level of ubiquitination per Fpn is elevated after 1 h and remains constant over time, suggesting Fpn is continuously ubiquitinated during holo-Tf-exposure.

### Apo- and holo-Tf differentially interact with Fpn and Heph

**Fig. 2:**
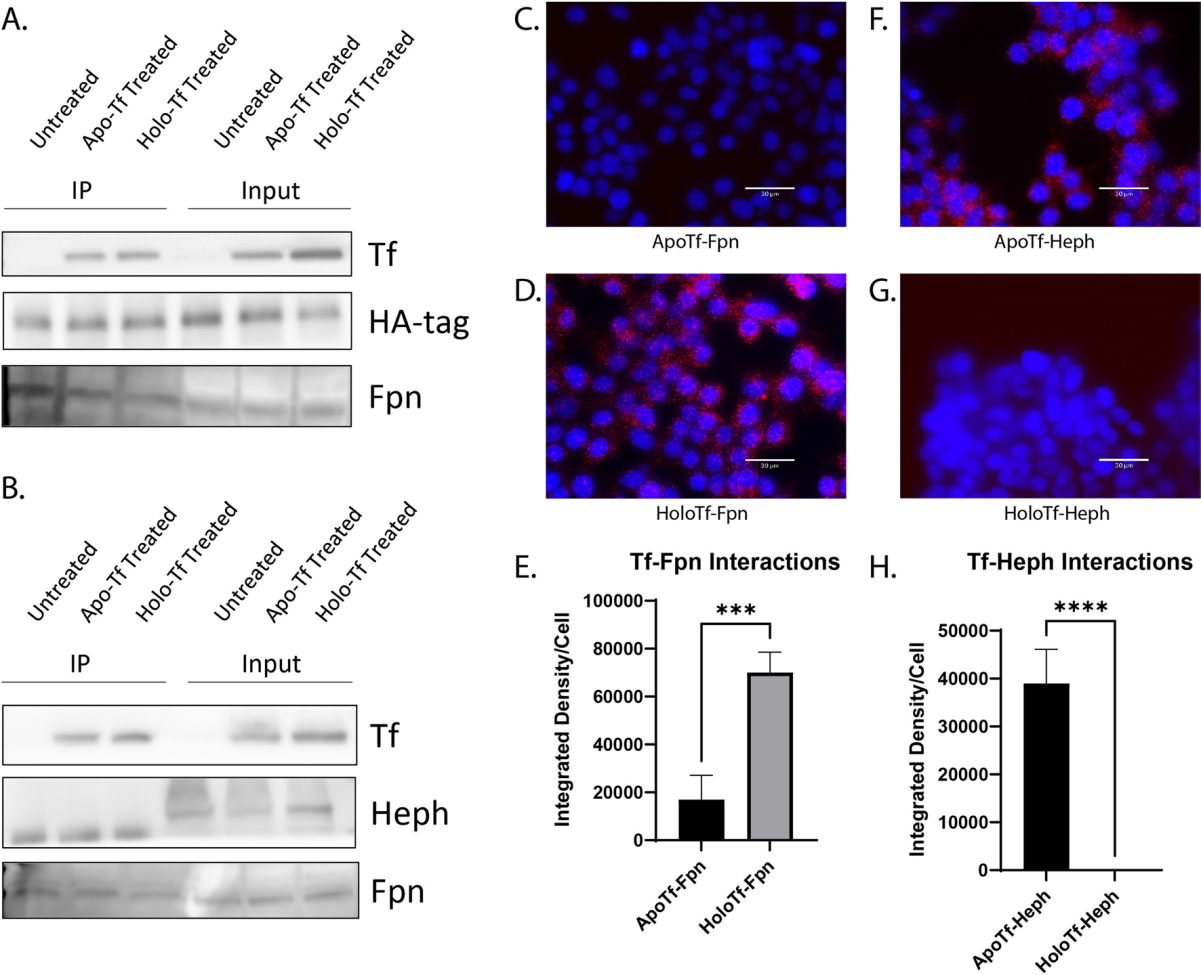
Apo- and holo-Tf interactions with Fpn and Heph. HEK 293 cells were transfected with HA-tagged Fpn and subsequently incubated with 0.25 μM apo- or holo-Tf for 10 min. Immunoprecipitate (IP) and 50% of cell lysate (input) was processed for immunoblotting. Co-IP of HA-Fpn shows that both apo- and holo-Tf are pulled down along with the Fpn complex (**A**). Co-IP of Heph in iPSC-derived ECs replicated these data (**B**). HEK 293 cells were used to determine direct protein interactions using PLA, reported as integrated density per cell in the field of view per image. Holo-Tf interacts with Fpn (**D**), while apo-Tf does not (**C**). Alternatively, apo-Tf interacts with Heph (**F**), while holo-Tf does not (**G**). n = 4 for all experiments, means of biological replicates ± SEM were evaluated for statistical significance using unpaired t test. ***p < 0.001, ****p < 0.0001

We next aimed to determine if holo-Tf interacted directly with Fpn. Due to their transfectability [26] and wide use for foundational biochemical studies [27, 28], as well as their universal iron export mechanism [24, 29, 30], we used HEK 293 cells transfected with an HA-tagged Fpn plasmid to selectively pull-down HA-Fpn. We incubated the cells with 0.25 μM of either apo- or holo-Tf (physiological level in CSF [25]) in media containing no FBS for 10 min prior to co-immunoprecipitation (co-IP). Regardless of whether the cells were incubated with either apo- or holo-Tf, Tf was co-immunoprecipitated with HA-Fpn (Fig. 2A). This indicates that apo- and holo-Tf bind to the Fpn complex of proteins. Because Heph aids Fpn in the export of iron [11], we hypothesized that apo-Tf could bind to Heph, leading to its co-immunoprecipitation with HA-Fpn. To confirm this possibility, we incubated ECs, which have greater Heph expression than HEK 293 cells, with either apo- of holo-Tf, and performed co-IP with Heph antibody. Again, in cells incubated with either apo- of holo-Tf, Tf was co-immunoprecipitated (Fig. 2B) further confirming that Fpn, Heph, and Tf complex together.

Because co-IP precipitates the entire complex of Fpn, Heph, apo-Tf, and holo-Tf, we aimed to better differentiate if apo- and holo-Tf directly interact with Fpn and Heph by employing proximity ligation assay (PLA), a highly sensitive method of detecting protein–protein interactions. ECs are highly polarized and exclusively express Fpn on the basolateral membrane [19, 31] that is adherent to the plate or slide surface, making it difficult to study protein interactions in this location. For this reason, we used HEK 293 cells for the PLA studies. Because the physiological concentration of Tf in the CSF is 0.25 μM [25], we chose this concentration for our studies. However, a range of concentrations of Tf were tested and no difference in PLA signal were found (Additional file 1: Fig. S2). All Tf incubations were in media containing no FBS for 10 min. Cells incubated with holo-Tf showed PLA signal when probing for a Tf and Fpn interaction (Fig. 2D), while cells incubated with apo-Tf showed PLA signal when probing for a Tf and Heph interactions (Fig. 2F). A small amount of PLA puncta just above background signal was detected when cells were treated with apo-Tf and probing for the Tf-Fpn interaction, however, this is likely due to apo-Tf binding to iron in the media and being converted to holo-Tf. Thus, holo-Tf directly interacts with Fpn while apo-Tf does not (***p < 0.001, Fig. 2D, E). Conversely, apo-Tf directly interacts with Heph, while holo-Tf does not (****p < 0.0001, Fig. 2F–H).

### High levels of hepcidin interrupt the interaction between holo-Tf and Fpn

**Fig. 3:**
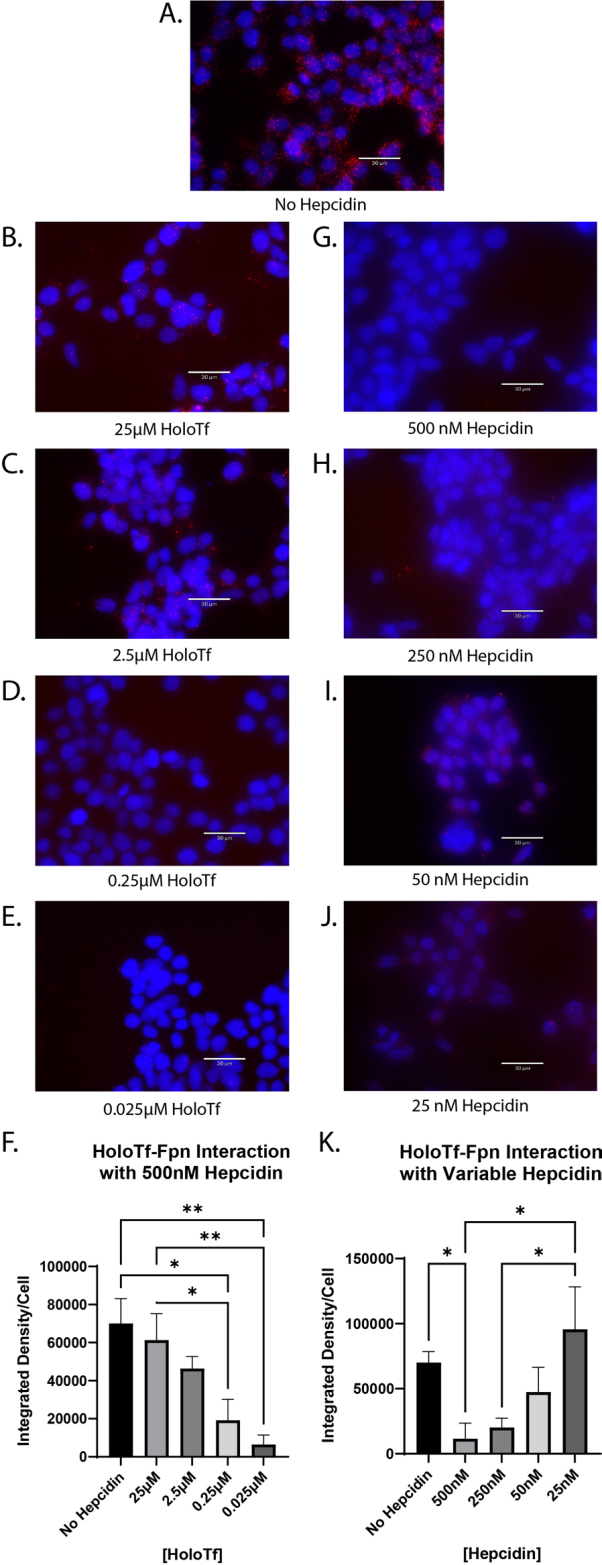
Hepcidin impact on interaction between holo-Tf and Fpn. HEK 293 cells were used to determine the impact of hepcidin on holo-Tf and Fpn interactions using PLA, reported as integrated density per cell in the field of view per image. The level of disrupted interaction was compared to a 0.25 μM holo-Tf and no hepcidin treatment control (**A**). Cells were co-incubated with holo-Tf and hepcidin for 10 min. The highest concentrations of hepcidin (500 nM) interrupt the interaction between holo-Tf and Fpn when holo-Tf is present at physiological (0.25 μM) levels (**D** and **G**), but not at the higher concentrations of holo-Tf concentrations (25 and 2.5 μM) (**B** and **C**) or when hepcidin concentrations are closer to physiological baseline of 25 nM (**H**–**J**). n = 3 for all experiments, means of biological replicates ± SEM were evaluated for statistical significance using one- way ANOVA with Tukey’s post-test for significance. *p < 0.05, **p < 0.01

Hepcidin is a well-known regulator and binding partner of Fpn, therefore we aimed to understand how the novel interaction between holo-Tf and Fpn could be impacted by physiological conditions that contribute to iron release. To do so, we used PLA to examine if hepcidin competed with holo-Tf for binding to Fpn. HEK 293 cells were co-incubated with 500 nM hepcidin (standard concentration in the literature [12, 15, 24]) and varying concentrations of holo-Tf (Fig. 3A–F) for 10 min. All co-incubation conditions were compared to the incubation with 0.25 μM holo-Tf (Fig. 3A). Hepcidin interrupted the interaction between 0.25 μM holo-Tf and Fpn (Fig. 3D), resulting in an 75% reduction of PLA signal (*p < 0.05) compared to no hepcidin treatment. Hepcidin was able to reduce the PLA signal by nearly 90% when the concentration of holo-Tf was only 0.025 μM (**p < 0.01, Fig. 3E). When holo-Tf was present in higher concentrations (25 μM and 2.5 μM), hepcidin did not interrupt the interactions between holo-Tf and Fpn (Fig. 3B, C, F), but these concentrations of holo-Tf are likely above physiological [32].

To determine if the amount of hepcidin was crucial to the interruption of the holo-Tf and Fpn interaction, we performed the reverse competition experiment and co-incubated HEK 293 cells with 0.25 μM holo-Tf and varying concentrations of hepcidin (Fig. 3G–K) for 10 min. Hepcidin interrupted the interaction between holo-Tf and Fpn in a dose dependent manner. The highest concentration of 500 nM significantly interrupted the interaction between holo-Tf and Fpn (*p < 0.05, Fig. 3G). However, the physiological concentration of hepcidin [33], 25 nM, had no impact on the holo-Tf-Fpn interaction (Fig. 3J).

### Hepcidin does not interrupt the interaction between apo-Tf and Heph

**Fig. 4:**
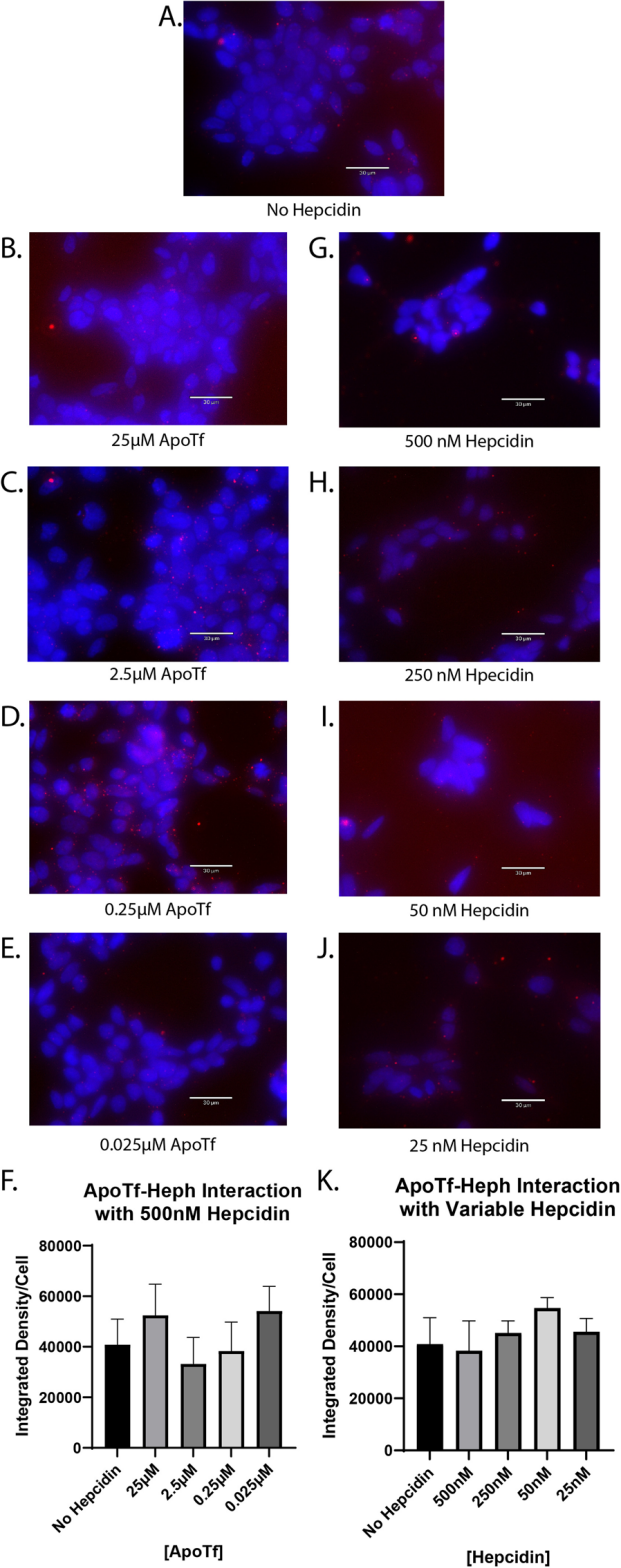
Hepcidin impact on interaction between apo-Tf and Heph. HEK 293 cells were used to determine the impact of hepcidin on apo-Tf and Heph interactions using PLA, reported as integrated density per cell in the field of view per image. Cells were co-incubated with apo-Tf and hepcidin for 10 min. The level of disrupted interaction was compared to a 0.25 μM apo-Tf and no hepcidin treatment control (**A**). Hepcidin has no impact on the interaction between apo-Tf and Heph at any apo-Tf concentrations (**B**–**E**) or at any hepcidin concentrations (**G**–**J**). n = 3 for all experiments, means of biological replicates ± SEM were evaluated for statistical significance using one- way ANOVA with Tukey’s post-test for significance

Apo-Tf has been shown to stimulate iron release despite the presence of hepcidin [4], thus we hypothesized that hepcidin would have no impact on the interaction between apo-Tf and Heph using PLA. HEK 293 cells were co-incubated with 500 nM hepcidin and varying concentrations of apo-Tf (Fig. 4B–F) for 10 min. Unlike with holo-Tf, 500 nM hepcidin did not interrupt the interaction between apo-Tf and Heph (Fig. 4B–E), as indicated by the unchanged PLA signal. In the reverse competition experiment, we co-incubated HEK 293 cells with 0.25 μM apo-Tf and varying concentrations of hepcidin (Fig. 4G–K). No concentration of hepcidin was sufficient to alter the interaction between apo-Tf and Heph (Fig. 4G–J). These data are consistent with previous findings that apo-Tf stimulates iron release from ECs even when co-incubated with hepcidin.

### Hepcidin internalizes Fpn faster than holo-Tf

**Fig. 5:**
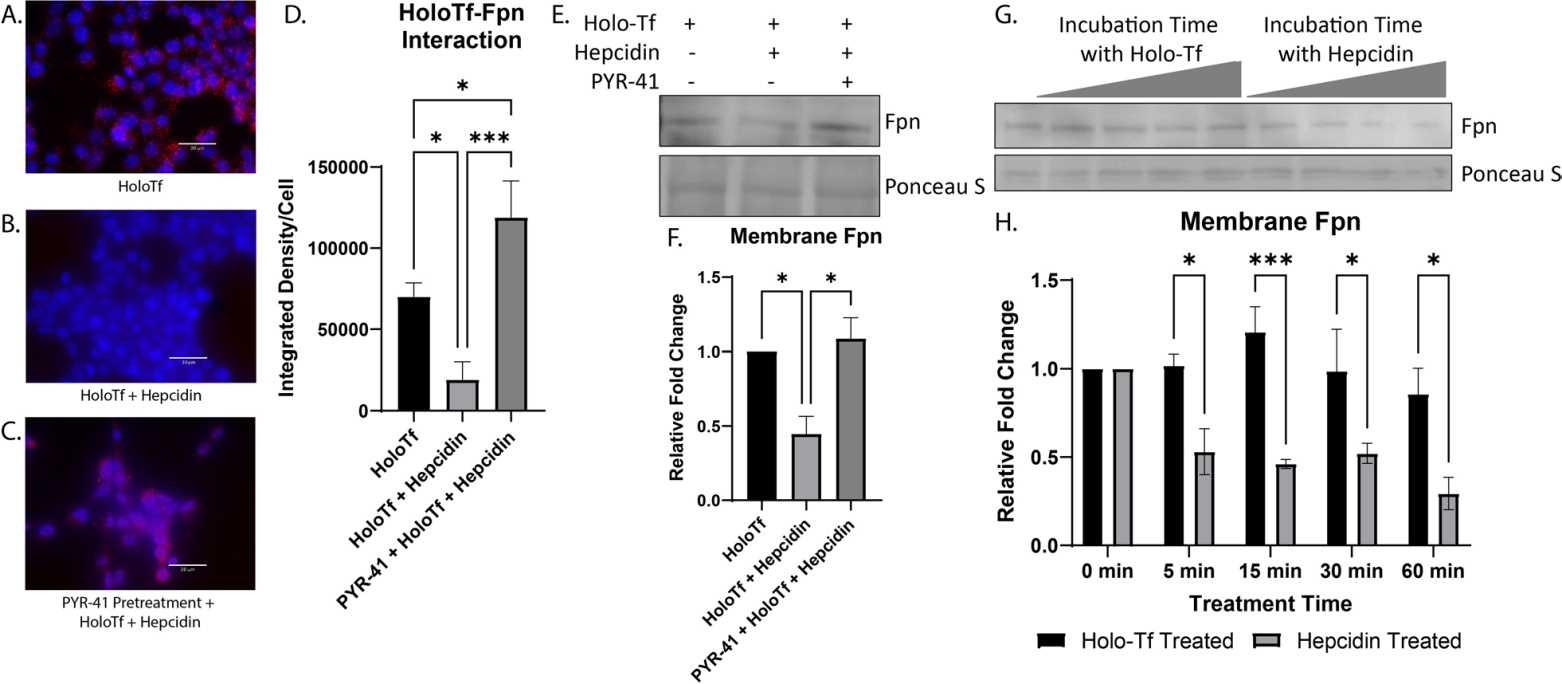
Modulation of Fpn internalization by hepcidin and holo-Tf. HEK 293 cells were used to determine the dynamics of holo-Tf and hepcidin on Fpn internalization using PLA, reported as integrated density per cell in the field of view per image (**A**–**D**). Pretreatment with PYR-41 (**C**) prevented the hepcidin induced reduction of interaction between holo-Tf and Fpn (**B**). The isolation of membrane bound Fpn confirms that hepcidin and holo-Tf co-incubation greatly reduces membrane Fpn levels, and this is prevented with PYR-41 (**E**–**F**). Hepcidin reduces membrane Fpn at a faster rate than holo-Tf (**G**, **H**). n = 3 to 5 for all experiments, means of biological replicates ± SEM were evaluated for statistical significance using one- way ANOVA with Tukey’s posttest for significance (**D**) and (**F**) or two-way ANOVA with Sidak’s post-test for significance (**H**). *p < 0.05, ***p < 0.001

The PLA experiments showed there was competition between holo-Tf and hepcidin, but did not differentiate between the possibility that either hepcidin was directly competing with holo-Tf for a binding site on Fpn or that hepcidin was internalizing Fpn faster than holo-Tf. To answer these questions, we utilized pretreatment with PYR-41, which prevents the degradation of Fpn and thus removes internalization dynamics as a factor in the binding of holo-Tf and hepcidin to Fpn. We performed PLA on HEK 293 cells exposed to 0.25 μM holo-Tf alone (Fig. 5A), 0.25 μM holo-Tf and 500 nM hepcidin for 10 min (Fig. 5B), and pretreatment of 50 μM PYR-41 for 30 min and then 0.25 μM holo-Tf and 500 nM hepcidin for 10 min (Fig. 5C). As reported in the experiments shown in Fig. 3D, hepcidin interrupts the interaction between holo-Tf and Fpn (*p < 0.05), however, this decreased interaction is prevented by PYR-41 pretreatment (***p < 0.001). This finding indicates that hepcidin decreases the interaction between holo-Tf and Fpn due to the ability of hepcidin to rapidly internalize Fpn. To further confirm a decrease of Fpn membrane presence by holo-Tf and hepcidin, we isolated membrane bound proteins following co-incubation of 0.25 μM holo-Tf and 500 nM hepcidin and found a significant decrease of membrane Fpn protein (*p < 0.05, Fig. 5E). This decrease in membrane Fpn is prevented when cells are pretreated with PYR-41 (*p < 0.01, Fig. 5E). These data align with the PLA results and suggests that hepcidin prevents holo-Tf from binding to Fpn by inducing the rapid internalization of Fpn.

The rate of Fpn internalization induced by holo-Tf or pathophysiological levels of hepcidin was further examined by incubating HEK 293 cells with either 0.25 μM holo-Tf or 500 nM hepcidin over time and subsequently isolating the membrane bound proteins. After only 5 min of 500 nM hepcidin incubation, membrane Fpn levels were decreased by nearly 50% compared to holo-Tf treatment (*p < 0.05, Fig. 5G). The trend continues at incubation times of 15 min (***p < 0.001), 30 min (*p < 0.05), and 60 min (*p < 0.05, Fig. 5G). By 60 min, hepcidin has internalized 70% of membrane Fpn compared to holo-Tf (*p < 0.05, Fig. 5G). By 60 min holo-Tf internalized 20% of Fpn compared to control (Fig. 5G).

## Discussion

This study addresses the molecular mechanisms by which apo- and holo-Tf regulate iron release from cells and provide insights to our previous findings at the BBB. More specifically, this study demonstrates that apo- and holo-Tf differentially interact with Heph and Fpn. Through its interaction with membrane bound Fpn, holo-Tf induces ubiquitination of and subsequent reduction in Fpn protein levels through the established Fpn degradation pathway. Holo-Tf directly interacts with Fpn as shown by orthogonal techniques of co-IP and PLA. Furthermore, when incubated together, high levels of hepcidin, that might correspond with inflammation or high systemic iron levels, can interrupt this interaction but not at baseline levels. The disruption in the holo-Tf and Fpn interaction by high concentrations of hepcidin appears to be due to hepcidin’s ability to internalize Fpn faster than holo-Tf and not due to direct competition for the same binding site. On the other hand, hepcidin does not interrupt the interaction between apo-Tf and Heph. These findings provide insights into the mechanism of free iron release into the brain and from cells in general. The discovery of the novel Tf protein interactions using both ECs and HEK 293 cells suggests that the mechanism may be applicable to general cellular iron export regulation, specifically for cells expressing hephaestin, which is most abundant in barrier cells [34].

Fpn is the only known iron exporter, thus control of the amount of membrane bound Fpn controls release of free iron. The internalization and subsequent degradation of Fpn has been extensively studied in the context of hepcidin [12, 15, 24, 35]. Briefly, once hepcidin binds to Fpn, it promptly triggers the ubiquitination of the Fpn, thus signaling for its internalization and lysosomal degradation. Simpson et al*.* showed that by incubating bovine retinal ECs (BRECs) with 12.5 μM holo-Tf, the levels of Fpn decreased [6]. Here, we have replicated those findings in iPSC-derived ECs but with physiological concentrations of Tf, which is found in CSF at about 2 mg/dL, or 0.25 μM [25]. We demonstrate that a concentration of holo-Tf as low as 0.1 μM results in a 50% decrease of membrane Fpn. These data provide a mechanistic explanation for why we have reported holo-Tf suppresses iron release from ECs [3, 6]. What’s more, other iron-related proteins, such as Heph, DMT1, and TfR, are relatively unchanged. Interestingly, even when exposed to high amounts of holo-Tf, the levels of Fpn do not decrease beyond 50%, suggesting there is a plateaued effect of holo-Tf within the 8-h experimental time window. The holo-Tf-mediated internalization of Fpn is blocked when the ubiquitination of Fpn is inhibited. Furthermore, incubation of 0.25 μM holo-Tf starts to induce Fpn ubiquitination within 1 h and peaks at about 3 h. Taken together, these data suggest that holo-Tf exerts its effect through the established degradation pathway, similar to hepcidin. Interestingly, the binding of hepcidin to Fpn immediately results in Fpn ubiquitination [24], whereas the binding of holo-Tf to Fpn seems to have a delayed ubiquitin response. We hypothesize that holo-Tf physically blocks the function of Fpn, causing an internal cellular mechanism to tag a seemingly faulty Fpn for degradation.

To complete the process of iron export from the endothelial cells, Fpn interacts with a complex of proteins, including Heph [10, 11]. Heph is a ferroxidase primarily expressed in barrier cells, such as ECs and enterocytes [34], that converts the Fpn-exported ferrous (Fe2+) to ferric (Fe3) that can bind to apo-Tf and be utilized by cells. Numerous studies have shown that Heph is required to stabilize Fpn in the plasma membrane and to enable iron export [10, 11, 36, 37]. We have replicated these findings, by demonstrating that Fpn and Heph can be co-immunoprecipitated from ECs. Furthermore, we demonstrate the novel finding that both apo- and holo-Tf independently are co-immunoprecipitated with Fpn and Heph. These results suggest that apo- and holo-Tf bind to Fpn and Heph in a complex of iron export proteins. In order to narrow down which protein holo-Tf bound to in the membrane that resulted in decreasing Fpn, we employed PLA. We found that holo-Tf directly interacts with Fpn, while apo-Tf does not. On the other hand, apo-Tf interacts with Heph, while holo-Tf does not, a finding that is supported in the literature [16, 18, 38]. It is hypothesized that apo-Tf binds to Heph to accept the ferric iron that Heph converts from ferrous iron. This stimulates the release of more iron as long as there is apo-Tf to accept it. Taken together these data suggest that apo- and holo-Tf differentially interact with iron export proteins, likely due to their structural differences [39]. The exact binding sites, conformation changes, and catalysts for these interactions are an exciting unexplored area that could pave the way for clinical manipulation. For example, as has been done experimentally [7], Tf could be infused to modulate iron accumulation in diseases in which it is dysregulated. Additionally, pharmaceuticals could be designed to facilitate or inhibit the endogenous protein interactions in an effort to correct brain iron accumulation.

Prior to the discovery that elevated holo-Tf could suppress iron release, hepcidin was the primary focus of iron release regulation [13]. Hepcidin is a pro inflammatory hormone peptide primarily secreted by the liver and upregulated in environments of inflammation and high iron levels [40]. Astrocytes [41, 42] and the choroid plexus [43, 44] have also been shown to secrete hepcidin, though in much smaller amounts that cannot account for total brain hepcidin levels [44, 45], suggesting much of the brain hepcidin comes from systemic levels when pathologically necessary, though this has not yet been proven. A number of groups have shown that astrocytic hepcidin reduces Fpn levels and subsequent iron release [14, 46, 47]. However, we have previously demonstrated that pathophysiological levels of hepcidin are not capable of blocking iron release from ECs [3, 4]. These data suggest that hepcidin cannot be the sole regulator of iron release in the brain. In support of this notion, Enculescu et al*.* modeled iron levels, and when compared to their experimental results, the study found that hepcidin control over iron uptake was necessary, but not sufficient [48]. Once a secondary regulatory mechanism was added to the model, their experimental results aligned with the model [48]. Thus, our data directly support that hepcidin is not the sole regulator of iron release and indicate the additional regulators are apo- and holo-Tf.

Our data offer an opportunity to explore the concept of regulation of iron uptake in general by hepcidin. We found that hepcidin competes with holo-Tf for binding to Fpn at low holo-Tf and high, pathophysiological hepcidin concentrations. However, when there was more holo-Tf or less hepcidin present, this effect was reduced. Notably, when hepcidin was only present at physiological levels [33], there was no interruption of the interaction between holo-Tf and Fpn. These findings suggest that hepcidin is only effective at controlling Fpn at levels consistent with inflammation or high iron. In observing competition between holo-Tf and hepcidin for Fpn binding, the internalization of Fpn was inhibited to determine if the competition was for binding site availability or rate of internalization. By preventing the internalization of Fpn, hepcidin had no impact on the interaction between holo-Tf and Fpn. This suggests that hepcidin internalizes Fpn faster than holo-Tf, which was confirmed by isolating membrane Fpn. Hepcidin reduces membrane Fpn by nearly 50% in 5 min, whereas holo-Tf only reduces membrane Fpn by 20% after 60 min. This finding is supported by Wallace et al*.* that showed hepcidin internalizes 50% of Fpn within 10 minutes [35]. In regard to the physical interaction between hepcidin, holo-Tf, and Fpn, the N-terminus of hepcidin is essential [49] for binding to the divalent metal Fpn C domain, resulting in the occlusion of iron efflux [50]. Based on our PLA data that show when the internalization of Fpn is prevented, there is no reduced interaction between holo-Tf and hepcidin, we suggest that the binding site for holo-Tf is different than that for hepcidin. If holo-Tf and hepcidin competed for the same binding site, we would have expected reduced PLA puncta. In contrast to the interaction between holo-Tf and Fpn, no amount of hepcidin impacts the interaction between apo-Tf and Heph. These data offer the intriguing suggestion that if apo-Tf is present, it will bind to Heph even in pathophysiological states and may be an explanation for iron accumulation in neurodegenerative disease. It has been postulated that in Alzheimer’s disease [51] and Parkinson’s disease [52], the brain may start as functionally iron deficient, along with elevated levels of apo-Tf, which triggers increased iron uptake until the excess iron detrimentally damages the BBB and surrounding cells. The question remains however, if the binding of apo-Tf to Heph will continue to stimulate iron release in the presence of hepcidin.

The model of apo- and holo-Tf regulation of iron release from ECs is a feedback loop. As cells, such as neurons or astrocytes, need iron for metabolic processes, myelin synthesis, or dopamine synthesis, they take up holo-Tf through TfR [53]. Once endocytosed, the iron is removed and the resulting apo-Tf is released [53]. The communication of brain iron status via apo- and holo-Tf allows cells to signal their iron needs based on their iron consumption. Numerous studies have shown higher regional iron uptake that correspond to areas with higher iron needs [8, 9, 54]. Our previous data suggest that as the apo- to holo-Tf ratio changes in the extracellular fluid, more iron is released locally from the BBB. In support of this notion are data showing CSF from iron deficient monkeys and iron chelated astrocytes increase iron release from cultured BRECs, while iron loaded biological samples resulted in decreased iron release [6]. These data have been replicated when cells are exposed to apo- or holo-Tf directly [3, 4, 6] or when apo- or holo-Tf is directly infused into the brain [7]. In all studies mentioned here, apo-Tf increased iron release while holo-Tf decreased iron release.

**Fig. 6:**
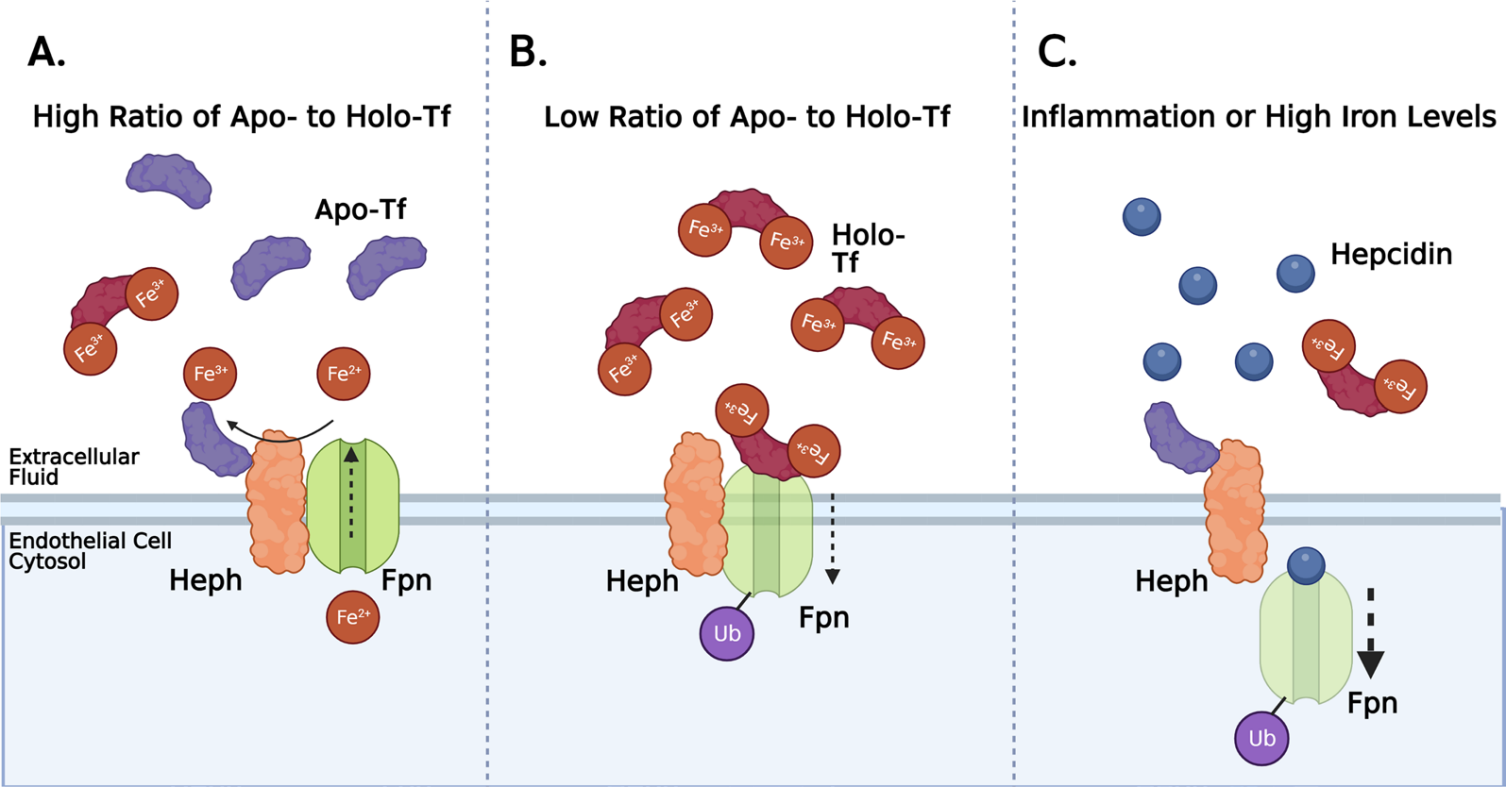
Model of Iron Release Regulation. In our proposed model, in areas that have higher ratios of apo- to holo-Tf (**A**), apo-Tf binds to Heph in order to accept the exported free iron and further stimulates iron release through Fpn. Alternatively, areas of lower ratios of apo- to holo-Tf (**B**), excessive holo-Tf binds to Fpn to facilitate the ubiquitination, internalization, and degradation of Fpn, and thus suppressing iron release through Fpn. In environments of inflammation or high iron levels, hepcidin production is upregulated (**C**). Hepcidin binds to Fpn and rapidly triggers Fpn’s internalization and abruptly stops free iron release

The data in this study expand the model for brain iron uptake by suggesting that apo-Tf stimulates iron release by binding to Heph to access exported free iron (Fig. 6A). Once loaded with iron, the now holo-Tf becomes available to surrounding cells. If the levels of holo-Tf in the extracellular fluid rise, holo-Tf binds to Fpn to suppress more iron release (Fig. 6B). The internalization of Fpn by holo-Tf is not rapid, unlike hepcidin. When upregulated and present in high amounts, hepcidin can rapidly internalize Fpn (Fig. 6C). Thus, we propose that hepcidin is likely used as a fast acting, immediate stop to iron release in environments of inflammation and very high iron. However, for moment-by-moment regional control of iron release, holo-Tf may be a better candidate to regulate regional iron supply.

## Conclusions

The regulation of brain iron uptake is not influenced by systemic levels [55], thus the regulation appears to come from the brain. Moreover, there are regional differences in the amount of iron in the brain. The data herein provide insights into a local regulatory process. This study is the first demonstration that apo- and holo-Tf differentially interact with Fpn and Heph to regulate iron release from ECs of the BBB. Moreover, we have identified a physiologically relevant dynamic between hepcidin and holo-Tf and their influence on membrane Fpn levels. Hepcidin interrupts the interaction between holo-Tf and Fpn by internalizing Fpn much faster than holo-Tf. Furthermore, we show that hepcidin does not interrupt the interaction between apo-Tf and hepcidin. These data suggest the mechanism of free iron release from ECs at the BBB that is likely relevant to cellular iron release in general. These results provide guidelines for further studies in neurological disease models where disruption in the iron regulatory mechanism may be disrupted and may provide additional insights of iron regulation beyond the BBB.

## Supplementary Information

**Additional file 1: Figure S1.** HA-tagged Fpn Plasmid Map. **Figure S2.** PLA Controls. **Figure S3.** Additional Iron Regulatory Proteins with Holo-Tf Incubation and PYR-41 Validation

## Data Availability

Data sharing is not applicable to this article as no datasets were generated or analyzed during the current study.

## Abbreviations

Apo-Tf: iron free transferrin
BBB: Blood–brain barrier
co-IP: Co-immunoprecipitation
ECs: Endothelial cells
Fpn: Ferroportin
Heph: Hephaestin
Holo-Tf: iron bound transferrin
iPSCs: Induced pluripotent stem cells
PLA: Proximity ligation assay
Tf: Transferrin
TfR: Transferrin receptor
