# Supplementary material for: Apo- and holo-transferrin differentially interact with hephaestin and ferroportin in a novel mechanism of cellular iron release regulation
Source: J Biomed Sci. 2023 Jun 6;30:36. doi: 10.1186/s12929-023-00934-2 (PMC10243088; doi:10.1186/s12929-023-00934-2)
Supplement: Supplementary file 1 — Additional file 1: Figure S1. HA-tagged Fpn Plasmid Map. Figure S2. PLA Controls. Figure S3. Additional Iron Regulatory Proteins with Holo-Tf Incubation and PYR-41 Validation [file 12929_2023_934_MOESM1_ESM.docx]

# Supplemental Data:

##
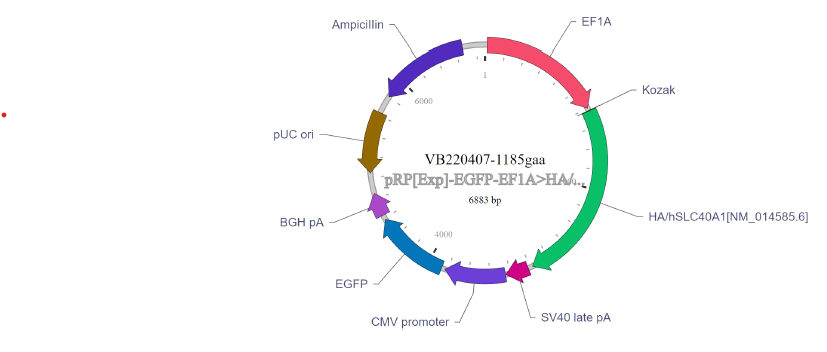
Supplemental Figure 1: HA-tagged Fpn Plasmid Map

HEK 293 cells were transfected with an HA-tagged Fpn plasmid in order to effectively pull down Fpn in co-IP experiments. The plasmid was designed using Vector Builder. The full sequence, as well as purchasing options, are available online <https://en.vectorbuilder.com/vector/VB220407-1185gaa.html>.

##
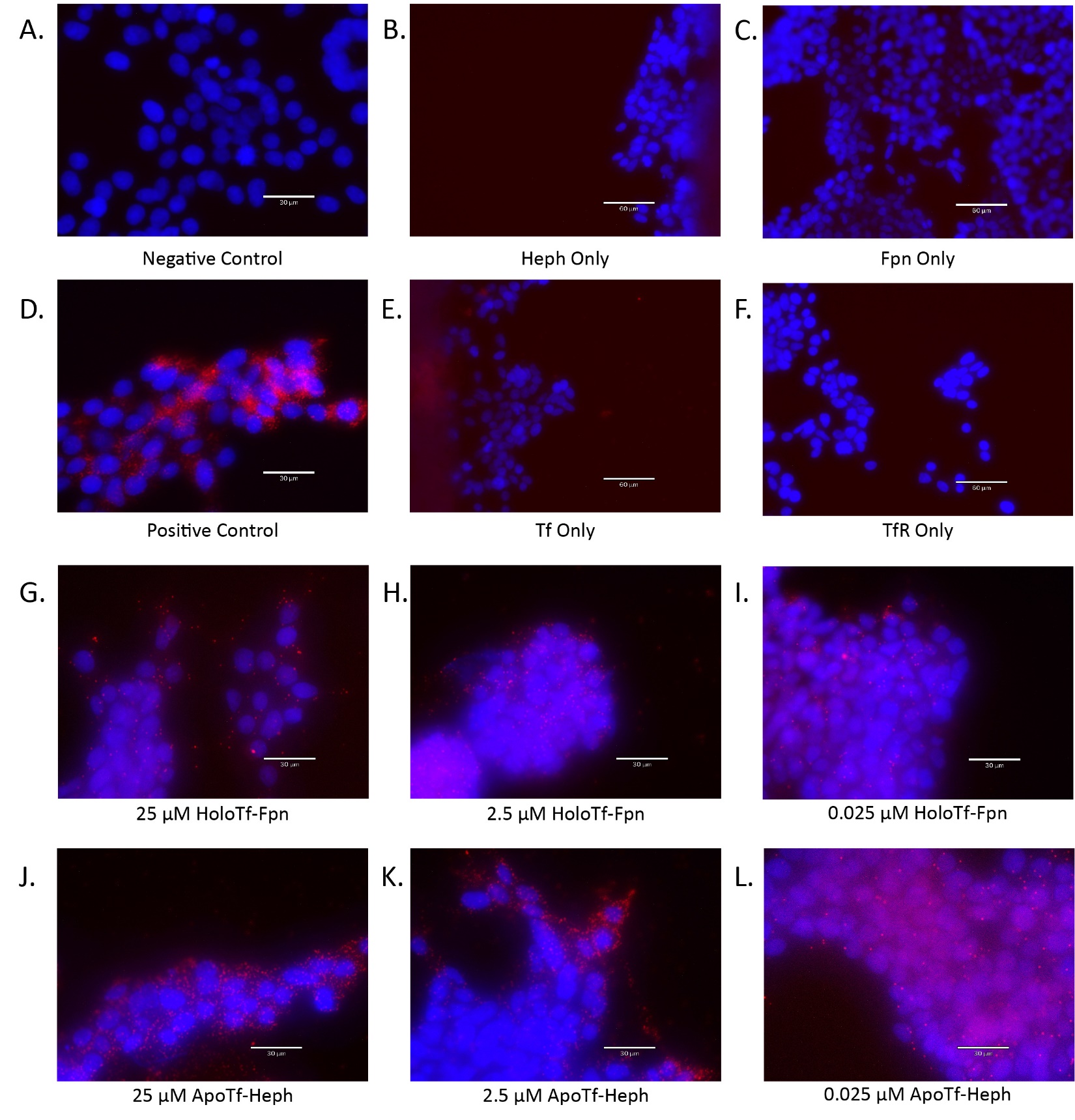
Supplemental Figure 2: PLA Controls

A number of controls were employed while optimizing the use of PLA to determine the interactions apo- and holo-Tf had with Heph and Fpn respectively. The negative control consisted of probing for two proteins known to not interact – here MBP and ferritin (**A**). The positive control consisted of probing for two proteins known to interact – here exogenous Tf and TfR (**D**). Furthermore, each antibody used to probe for Fpn, Heph, Tf, and TfR was assessed for nonspecific binding by performing solo incubations and ensuring the antibody alone did not produce PLA signal (**B-C, E-F**). In order to determine the best concentration of apo- and holo-Tf to use, we tested 0.025 μM, 2.5 μM, and 25 μM along with 0.25 μM (which was used for experiments) (**G-L**). All additional concentrations of apo- and holo-Tf show similar amounts of PLA puncta as the used 0.25 μM.

##
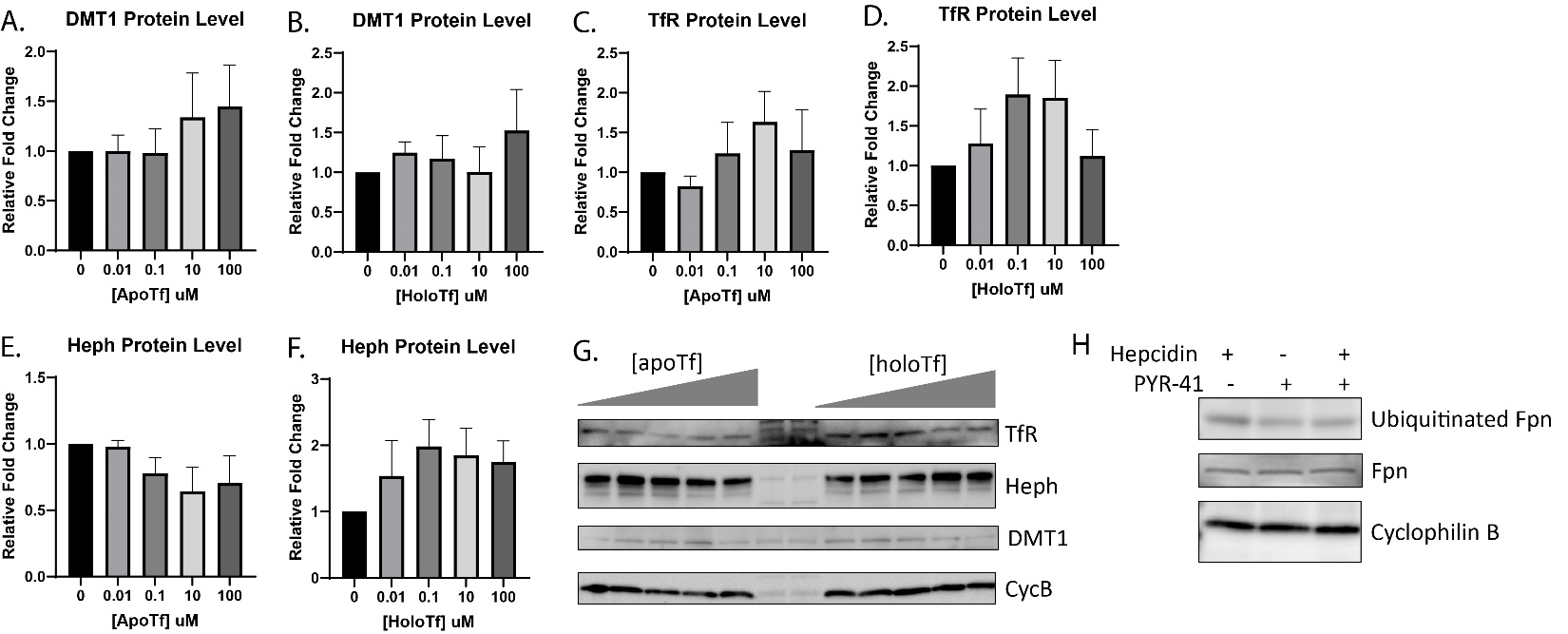
Supplemental Figure 3: Additional Iron Regulatory Proteins with Holo-Tf Incubation and PYR-41 Validation

iPSC-derived ECs were incubated with holo-Tf in the basal chamber of Transwell inserts. The cells were collected and additional iron regulatory proteins were probed for using immunoblotting. Neither apo- nor holo-Tf incubations resulted in significant changes to DMT1, Heph, or TfR protein levels (**A-G**). PYR-41’s inhibition of ubiquitination was validated using hepcidin to trigger Fpn ubiquitination (**H**). Exposure to hepcidin alone for 30 minutes increases ubiquitination of Fpn. When pretreated with PYR-41 for 30 minutes, the increased ubiquitination of Fpn is blocked. Total Fpn levels are unchanged. n=3 to 4 for all experiments, means of biological replicates ± SEM were evaluated for statistical significance using one- way ANOVA with Tukey’s posttest for significance.
